# Effectiveness of a Web-Based Individual Coping and Alcohol Intervention Program for Children of Parents With Alcohol Use Problems: Randomized Controlled Trial

**DOI:** 10.2196/52118

**Published:** 2024-04-10

**Authors:** Håkan Wall, Helena Hansson, Ulla Zetterlind, Pia Kvillemo, Tobias H Elgán

**Affiliations:** 1 Stockholm Prevents Alcohol and Drug Problems, Centre for Psychiatry Research Department of Clinical Neuroscience Karolinska Institutet, & Stockholm Health Care Services Stockholm Sweden; 2 School of Social Work Faculty of Social Sciences Lund University Lund Sweden; 3 Clinical Health Promotion Centre Department of Health Sciences Lund University Lund Sweden

**Keywords:** adolescent, adolescents, alcoholic, alcoholics, CBT, children of impaired parents, cognitive behavioral therapy, coping, digital intervention, mental health, randomized controlled trial, RCT, self-management, substance abuse, substance use, therapist, web-based intervention, youth

## Abstract

**Background:**

Children whose parents have alcohol use problems are at an increased risk of several negative consequences, such as poor school performance, an earlier onset of substance use, and poor mental health. Many would benefit from support programs, but the figures reveal that only a small proportion is reached by existing support. Digital interventions can provide readily accessible support and potentially reach a large number of children. Research on digital interventions aimed at this target group is scarce. We have developed a novel digital therapist-assisted self-management intervention targeting adolescents whose parents had alcohol use problems. This program aims to strengthen coping behaviors, improve mental health, and decrease alcohol consumption in adolescents.

**Objective:**

This study aims to examine the effectiveness of a novel web-based therapist-assisted self-management intervention for adolescents whose parents have alcohol use problems.

**Methods:**

Participants were recruited on the internet from social media and websites containing health-related information about adolescents. Possible participants were screened using the short version of the Children of Alcoholics Screening Test-6. Eligible participants were randomly allocated to either the intervention group (n=101) or the waitlist control group (n=103), and they were unblinded to the condition. The assessments, all self-assessed, consisted of a baseline and 2 follow-ups after 2 and 6 months. The primary outcome was the Coping With Parents Abuse Questionnaire (CPAQ), and secondary outcomes were the Center for Epidemiological Studies Depression Scale, Alcohol Use Disorders Identification Test (AUDIT-C), and Ladder of Life (LoL).

**Results:**

For the primary outcome, CPAQ, a small but inconclusive treatment effect was observed (Cohen *d*=–0.05 at both follow-up time points). The intervention group scored 38% and 46% lower than the control group on the continuous part of the AUDIT-C at the 2- and 6-month follow-up, respectively. All other between-group comparisons were inconclusive at either follow-up time point. Adherence was low, as only 24% (24/101) of the participants in the intervention group completed the intervention.

**Conclusions:**

The findings were inconclusive for the primary outcome but demonstrate that a digital therapist-assisted self-management intervention may contribute to a reduction in alcohol consumption. These results highlight the potential for digital interventions to reach a vulnerable, hard-to-reach group of adolescents but underscore the need to develop more engaging support interventions to increase adherence.

**Trial Registration:**

ISRCTN Registry ISRCTN41545712; https://www.isrctn.com/ISRCTN41545712?q=ISRCTN41545712

**International Registered Report Identifier (IRRID):**

RR2-10.1186/1471-2458-12-35

## Introduction

Children who grow up with parents who have substance use problems or disorders face extraordinary challenges. Approximately 20% of all children have parents with alcohol problems [1-5], while approximately 5% have parents with alcohol use disorders [4,6,7]. Children growing up with parental substance abuse are at an increased risk of several negative outcomes, such as psychiatric morbidity [8-12]; poor intellectual, cognitive, and academic achievement [13-15]; domestic physical abuse [16]; and early drinking onset and the development of substance use problems [9,17,18]. Thus, children exposed to parental substance abuse comprise a target group for selective interventions and prevention strategies [19-22].

In Sweden, municipalities account for most of the support offered to these children. An annual survey by the junior association of the Swedish branch of Movendi International (ie, an international temperance movement) reported that 97% of all municipalities provided support resources [23]. However, estimates from the same survey showed that approximately 2% of the children in the target group received support. Hence, an overwhelming majority never receives support, mainly because of difficulties in identifying and attracting them to intervention programs [22,24].

The internet has become an appealing way to reach and support a large number of people [25,26]. Web-based interventions seem particularly attractive to adolescents, as they generally use digital technology and social media. Furthermore, research has shown that adolescents regard the internet as inviting because it is a readily accessible, anonymous way of seeking help [27]. Web-based interventions can reduce the stigma associated with face-to-face consultations in health care settings [28], and young people appreciate the flexibility of completing web-based sessions to fit their own schedules [29]. The positive effects of web-based interventions have been detected across a broad range of conditions. A recent review by Hedman-Lagerlöf et al [30] concluded that therapist-supported internet-based cognitive behavioral therapy for adults yielded similar effects as face-to-face therapy. To date, most web-based interventions have been designed for adults. Although the number of web-based interventions targeting children or adolescents is increasing [25,31-33], the number of digital interventions aimed at children of substance-abusing parents is still scarce [22,34-38]. Those described in the literature, however, all have in common that they are quite extensive, with a duration over several weeks, and a brief digital intervention could complement these more extended interventions. For instance, our research group initiated a study on a web-based group chat for 15- to 25-year-old individuals who have parents with mental illness or substance use problems [35]. The duration of the program is 8 weeks, and it is a translated version of a program from the Netherlands [34], which has been shown to have inconclusive treatment effects [39]. In Sweden, 2 other programs with inconclusive treatment effects have been tested that target significant others and their children [37,38]. Finally, a digital intervention developed in Australia for 18- to 25-year-old individuals with parents with mental illness or substance use disorder [36] was tested in a pilot study demonstrating positive findings [40].

To meet the need for a brief, web-based intervention that targets adolescents having parents with alcohol problems and build on the evidence base of digital interventions targeting this vulnerable group, we developed a novel internet-delivered therapist-assisted self-management intervention called “Alcohol and Coping.” Our program originated from a manual-based face-to-face intervention called the “Individual Coping and Alcohol Intervention Program” (ICAIP) [41,42]. Previous studies on both the ICAIP, which aimed at college students having parents with alcohol problems, and a coping skills intervention program, which aimed at spouses of partners with alcohol dependency [43], have demonstrated positive effects regarding decreased alcohol consumption and improved mental health and coping behaviors [41-44]. Furthermore, the results from these studies underscore the importance of improving coping skills [42,44]. Among college students, those who received a combination of coping skills and an alcohol intervention program had better long-term outcomes [42].

The aim of this study was to test the effectiveness of Alcohol and Coping among a sample of adolescents aged 15-19 years with at least 1 parent with alcohol use problems. We hypothesized that the intervention group would be superior to the control group in improving coping skills. Secondary research questions concerned the participants’ improvement in (1) depression, (2) alcohol consumption, and (3) quality of life.

## Methods

### Overview

**Figure 1:**
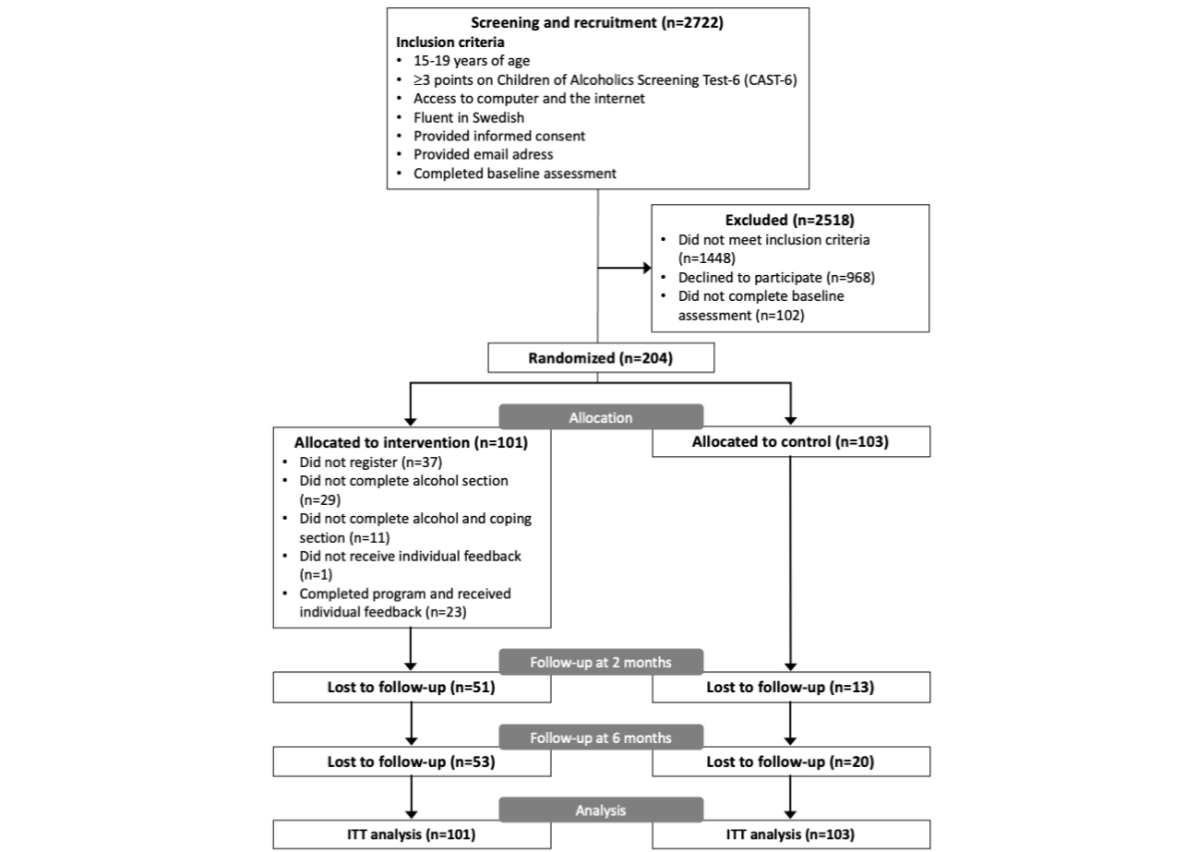
CONSORT (Consolidated Standards of Reporting Trials) diagram depicting study design and participant flow. ITT: intention-to-treat.

This study was a parallel-group randomized controlled trial in which participants were randomized to either the intervention or waitlist control group in a 1:1 allocation ratio. The trial design is illustrated in Figure 1.

### Recruitment and Screening

The participants were recruited from August 2012 to December 2013 through advertisements on social media (Facebook). The advertisements targeted individuals aged 15-19 years with Facebook accounts. Participants were recruited on the internet through advertisements on websites containing health-related information about adolescents. The advertisements included the text, “Do your parents drink too much? Participate in a study.” The advertisement contained an invitation to perform a web-based, self-assessed screening procedure. In addition to questions about age and sex, participants were screened for having parents with alcohol problems using the short version of the Children of Alcoholics Screening Test-6 (CAST-6), developed from a 30-item original version [45]. The CAST-6 is a 6-item true-false measure designed to assess whether participants perceive their parents’ alcohol consumption to be problematic. The CAST-6 has demonstrated high internal consistency (*r*=0.92-0.94), test-retest reliability (*r*=0.94), and high validity as compared to the 30-item version (*r*=0.93) using the recommended threshold score of 3 or higher [45,46]. We previously translated the CAST-6 into Swedish and validated the translated version among 1450 adolescents, showing good internal consistency (α=.88), excellent test-retest reliability (intraclass correlation coefficient=0.93), and loading into 1 latent factor [47]. Additional inclusion criteria included having access to a computer and the internet and being sufficiently fluent in Swedish. Participants were excluded from the study and were referred to appropriate care if there were indications of either suicidal or self-inflicted harmful behaviors. Individuals eligible for inclusion received further information about the study and were asked to provide consent to participate by providing an email address.

### Data Collection and Measures

All assessments were administered through email invitations containing a hyperlink to the web-based self-reported assessments. Up to 3 reminders were sent through email at 5, 10, and 15 days after the first invitation. A baseline assessment (t_0_) was collected before randomization, and follow-up assessments were conducted at 2 and 6 months (t_1_ and t_2_, respectively) after the initial assessment.

Participants were asked for age, sex, whether they lived with a parent (mother and father, mother or father, mother or father and stepparent, or alternate between mother and father), where their parents were born (Sweden or a Nordic country excluding Sweden or outside of the Nordic countries), parental status (employed, student, on parental leave, or unemployed), and any previous or present participation in support activities for children having parents with alcohol use problems. The primary outcome was coping, measured using the Coping With Parents Abuse Questionnaire (CPAQ) based on the Coping Behavior Scale developed by Orford et al [48]. Secondary outcomes were the Center for Epidemiological Studies Depression Scale (CES-DC) [49], the 3-question Alcohol Use Disorders Identification Test (AUDIT-C) [50], and the Ladder of Life (LoL), which measures the overall quality of life by asking about the participants’ past, present, and future ratings of their overall life satisfaction [50]. CPAQ has been shown to be reliable [41,42]. For this study, this scale was factor-analyzed to reduce the number of questions from 37 to 20. The resulting scale measures 6 coping typologies (discord, emotion, control, relationship, avoidance, and taking specific action) using a 4-point Likert scale, with a threshold score above 50 points (out of 80) indicating dysfunctional coping behavior. The CES-DC measures depressive symptoms during the past week using a 4-point Likert scale, where a higher total score indicates more depressive symptoms [49]. A cutoff score of ≥16 indicates symptoms of moderate depression, while a score of ≥30 indicates symptoms of severe depression [51,52]. The scale measures 4 dimensions of depression: depressed mood, tiredness, inability to concentrate, and feelings of being outside and lonely, and has positively stated items [52]. Additionally, this scale is a general measure of childhood psychopathology [53] and has been demonstrated to be reliable and valid among Swedish adolescents [52]. Alcohol consumption was measured using a modified AUDIT-C, which assesses the frequency of drinking, quantity consumed on a typical occasion, and frequency of heavy episodic drinking (ie, binge drinking) [50] using a 30-day perspective (as opposed to the original 12-month perspective). These questions have previously been translated into Swedish [54], and a score of ≥4 and ≥5 points for women and men, respectively, was used as a cutoff for risky drinking. This scale has been demonstrated to be reliable and valid for Swedish adolescents [55]. Furthermore, 2 questions were added concerning whether the participants had ever consumed alcohol to the point of intoxication and their age at the onset of drinking and intoxication. The original version of the LoL was designed for adults and asked the respondents to reflect on their, present, and future life status from a 5-year perspective on a 10-point Visual Analogue Scale representing life status from “worst” to “best” possible life imaginable [56]. A modified version for children, using a time frame of 1 year, has been used previously in Sweden [57] and was used in this study.

### Randomization

After completing the baseline assessment, each participant was allocated to either the intervention or the control group. An external researcher generated an unrestricted random allocation sequence using random allocation software [58]. Neither the participants nor the researchers involved in the study were blinded to group allocation.

### Procedure

Based on the order in which participants were included in the study, they were allocated to 1 of the 2 study groups and informed of their allocation by email. Additionally, those who were randomized to the intervention group received a hyperlink to the Alcohol and Coping program, whereas the control group participants received information that they would gain access to Alcohol and Coping after the last follow-up assessment (ie, the waitlist control group). All participants were informed about other information and support available through web pages, notably drugsmart [59], which contains general information and facts about alcohol and drugs, in addition to more specific information about having substance-abusing parents. Telephone numbers and contact information for other organizations and primary health care facilities were also provided.

### The Intervention

**Figure 2:**
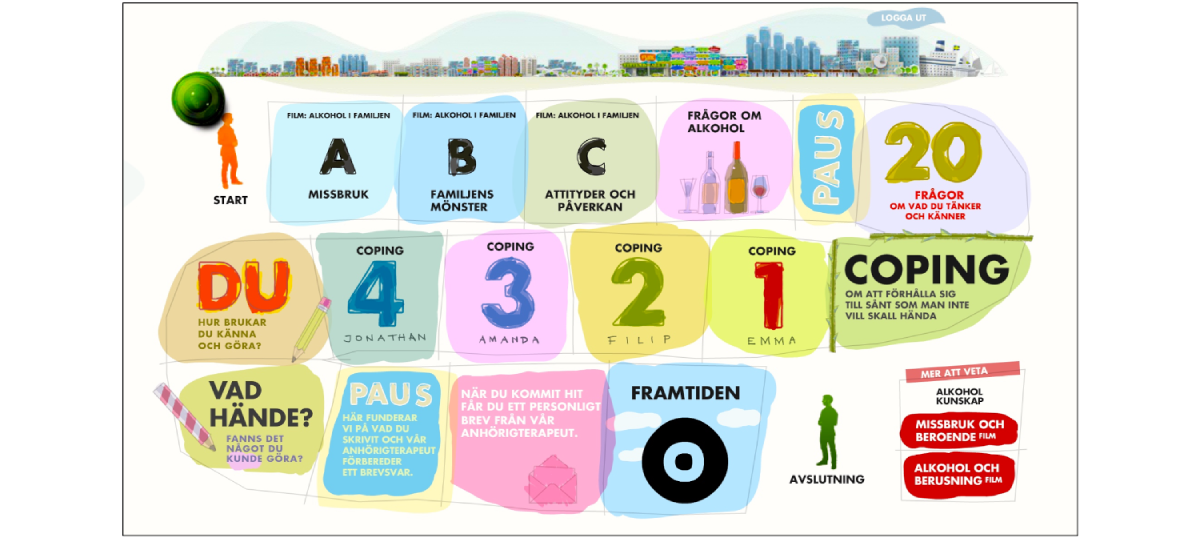
The procedure of Alcohol and Coping, which followed the pattern of a board game where participants needed to go through all parts of the program from start to end (Swedish, “avslutning”).

**Figure 3:**
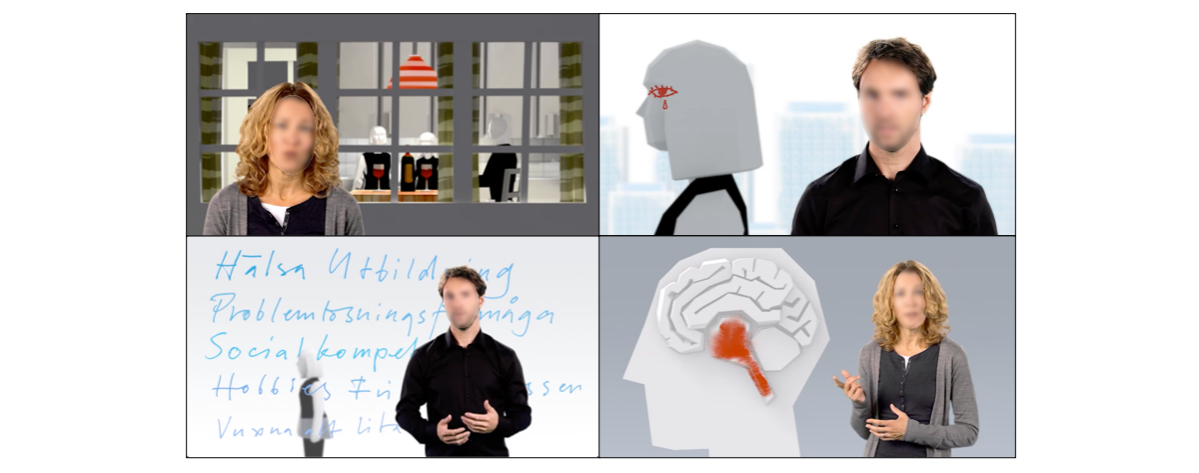
A total of 4 snapshots from the 4 film-based lectures on alcohol and coping concerning, for instance, alcohol problems and family patterns, emotion and problem-focused coping, and physiological effects of alcohol, intoxication, and addiction.

As noted previously, Alcohol and Coping is derived from the aforementioned manual-based face-to-face ICAIP intervention program [41,42]. The ICAIP consists of a combination of an alcohol intervention program, which is based on the short version of the Brief Alcohol Screening and Intervention for College Students program [60], and a coping intervention program developed for the purpose of the ICAIP [41,42]. Like the original ICAIP intervention, Alcohol and Coping builds on psychoeducational principles and includes components such as film-based lectures, various exercises, and both automated and therapist-assisted feedback. Briefly, once the participants logged into the Alcohol and Coping platform, they were introduced to the program, which followed the pattern of a board game (Figure 2). Following the introduction, participants took part in 3 film-based lectures (between 8 and 15 minutes each, Figure 3) concerning alcohol problems within the family. The respective lectures included information about (1) dependency in general as well as the genetic and environmental risks for developing dependency, (2) family patterns and how the family adapts to the one having alcohol problems, and (3) attitudes toward alcohol and how they influence drinking and the physiological effects of alcohol. After completing the lectures, the participants were asked to answer 2 questions about their own alcohol consumption (ie, how often they drink and how often they drink to intoxication), followed by an automatic feedback message that depended on their answers. It was then suggested that the participants log out of the intervention for a 1- to 2-day break. The reason for this break was to give the participants a chance to digest all information and impressions. When they logged back into the intervention, they were asked to answer 20 questions about their coping strategies, which were also followed by automatic feedback. This feedback comprised a library covering all the prewritten feedback messages, each of which was tailored to the participants’ specific answers. The participants then participated in a 5-minute–long film-based lecture on emotion and problem-focused coping in relation to family alcohol problems (Figure 3). This was followed by 4 exercises where the participants read through vignette-like stories from 4 fictional persons describing their everyday lives related to coping and alcohol problems in the family. The stories are presented by film-based introductions that are each 1-2 minutes long. Participants were then requested to respond to each story by describing how the fictive person could have coped with their situation. As a final exercise, participants were asked to reflect on their own family situation and how they cope with situations. The participants then had to take a break for a few days.

During the break, a therapist composed individual feedback that covered reflections and confirmation of the participant’s exercises and answers to questions and included suggestions on well-suited coping strategies. Additionally, the therapist encouraged the participants to talk to others in their surroundings, such as friends, teachers, or coaches, and seek further support elsewhere, such as from municipal social services, youth health care centers, or other organizations. Finally, the therapist reflected on the participants’ alcohol consumption patterns and reminded them of increased genetic and environmental risks. Those who revealed patterns of risky alcohol use were encouraged to look at 2 additional film-based lectures with more information about alcohol and intoxication (4 minutes) and alcohol use and dependency (5 minutes). Participants received this feedback once they logged back into the program, but they also had the opportunity to receive feedback through email. The total estimated effective time for completing the program was about 1 hour, but as described above, there was 1 required break when the individualized feedback was written. To keep track of the dose each participant received, each of the 15 components in the program (Figure 1) is equal to completing 6.7% (1/15) of the program in total.

### Sample Size

The trial was designed to detect a medium or large effect size corresponding to a standardized mean difference (Cohen *d* >0.5) [61]. An a priori calculation of the estimated sample size, using the software G*Power (G*Power Team) [62], revealed that a total of 128 participants (64 in each group) were required to enroll in the trial (power=0.80; α=.05; 2-tailed). However, to account for an estimated attrition rate of approximately 30% [34], it was necessary to enroll a minimum of 128/(1 – 0.3) = 183 participants in the trial. After a total of 204 individuals had been recruited and randomized into 2 study arms, recruitment was ended.

### Statistical Analysis

Data were analyzed according to the intention-to-treat (ITT) principle, and all randomized participants were included, irrespective of whether they participated in the trial. The 4 research variables were depression (CES-DC), coping (CPAQ), alcohol use (AUDIT-C), and life status (LoL).

Data analysis consisted of comparing outcome measurements at t_1_ and t_2_. The baseline measurement t_0_ value was added as an adjustment variable in all models. The resulting data from CPAQ, CES-DC, and LoL were normally distributed and analyzed using linear mixed models. The resulting AUDIT-C scores were nonnormally distributed, with an excess of 0 values, and were analyzed using a 2-part model for longitudinal data. This model is sufficiently flexible to account for numerous 0 reports. This was achieved by combining a logistic generalized linear mixed model (GLMM) for the 0 parts and a skewed continuous GLMM for the non-0 alcohol consumption parts. R-package *brms* (Bayesian regression models using Stan; R Foundation for Statistical Computing) [63], a higher-level interface for the probabilistic programming language Stan [64], and a custom *brms* family for a marginalized 2-part lognormal distribution were used to fit the model [65]. The logistic part of the model represents the subject-specific effects on the odds of reporting no drinking. The continuous part was modeled using a gamma GLMM with a log link. The exponentiated treatment effect represents the subject-specific ratio of the total AUDIT-C scores between the treatment and waitlist control groups for those who reported drinking during the specific follow-up period.

### Handling of Missing Data

GLMMs include all available data and provide unbiased ITT estimates under the assumption that data are missing at random, meaning that the missing data can be explained by existing data. However, it is impossible to determine whether the data are missing at random or whether the missing data are due to unobserved factors [66]. Therefore, we also assumed that data were not missing at random, and subsequent sensitivity analyses were performed [66]. We used the pattern mixture method, which assumes not missing at random, to compare those who completed the follow-up at 6 months (t_2_) with those who did not (but completed the 2-month follow-up). The overall effect of this model is a combination of the effects of each subgroup. We also tested the robustness of the results by performing ANCOVAs at the 2-month follow-up, both using complete cases and with missing values imputed using multilevel multiple imputation.

The effect of the program was estimated using Cohen *d*, where a value of approximately 0.2 indicates a small effect size and values of approximately 0.5 and 0.8 indicate medium and large effect sizes, respectively [61].

### Ethical Considerations

All procedures were performed in accordance with the ethical standards of the institutional or national research committees, the 1964 Helsinki Declaration and its later amendments, and comparable ethical standards. Informed consent was obtained from all the participants included in the study. This study was approved by the Swedish Ethical Review Authority (formerly the Regional Ethical Review Board in Stockholm, No. 2011/1648-31/5).

To enhance the response rates, participants received a cinema gift certificate corresponding to approximately EUR 11 (US $12) as compensation for completing each assessment. If a participant completed all assessments, an additional gift certificate was provided. The participants could subsequently receive 4 cinema gift certificates totaling EUR 44 (US $48).

## Results

### Overview

The trial profile is depicted in Figure 1 and reveals that 2722 individuals who were aged between 15 and 19 years performed the screening procedure. A total of 1448 individuals did not fulfill the inclusion criteria and were excluded, leaving 1274 eligible participants. Another 1070 individuals were excluded because they did not provide informed consent or complete the baseline assessment, leaving 204 participants who were allocated to 1 of the 2 study groups. A total of 140 (69%) and 131 (64%) participants completed t_1_ and t_2_ assessments, respectively. Of the participants in the intervention group (n=101), 63% (n=64) registered an account on the Alcohol and Coping website, 35% (n=35) completed the alcohol intervention section, and 24% (n=24) completed both the alcohol and coping intervention sections.

### Sample Characteristics

The mean age of the sample was 17.0 (SD 1.23) years, and the vast majority were female, with both parents born in Sweden and currently working (Table 1). Approximately one-third of the participants reported living with both parents. The mean score on the CAST-6 was 5.33 (SD 0.87) out of a total of 6, and the majority of the sample (147/204, 72.1%) perceived their father to have alcohol problems. Approximately 12% (25/204) had never consumed alcohol, whereas approximately 70% (144/204) had consumed alcohol at a level of intoxication. The mean age at onset was 13.7 (SD 2.07) years and the age at first intoxication was 14.8 (SD 1.56) years. The proportion of participants with symptoms of at least moderate depression was 77.5% (158/204), of whom 55.1% (87/158) had symptoms of severe depression and 42.6% (87/204) had symptoms of dysfunctional coping behaviors. The percentage of participants who consumed alcohol at a risky level was 39.7% (81/204). Table 1 provides complete information regarding the study sample.

**Table 1:** Participant demographics at baseline.

		Intervention (n=101)	Control (n=103)	*P* value^a^	
Age (years), mean (SD)		17.0 (1.29)	16.9 (1.18)	.74	
Sex (female), n (%)		87 (86.1)	92 (89.3)	.49	
Children of Alcoholics Screening Test-6 points, mean (SD)		5.45 (.82)	5.23 (.91)	.08	
**Family member with perceived alcohol problems, n (%)**					
	Mother	28 (27.7)	35 (34)	.33	
	Father	75 (74.3)	72 (69.9)	.49	
	Stepmother	8 (7.9)	7 (6.8)	.76	
	Stepfather	10 (9.9)	14 (13.6)	.41	
	Other	5 (5)	6 (5.8)	.78	
**I live with, n (%)**					.71
	Mother and father	29 (28.7)	29 (28.2)		
	Mother or father	20 (19.8)	25 (24.3)		
	Mother, father and stepmother, or father	16 (15.8)	20 (19.4)		
	Part-time with mother and father	13 (12.9)	13 (12.6)		
	I do not live with any of my parents	16 (15.8)	9 (8.7)		
**Mother was born in, n (%)**					
	Sweden	90 (90.1)	83 (80.6)	.06	
	Other Nordic country	4 (4)	9 (8.7)	.16	
	Outside Nordic country	6 (5.9)	12 (11.7)	.15	
**Father was born in, n (%)**					
	Sweden	84 (83.2)	84 (81.6)	.76	
	Other Nordic country	6 (5.9)	9 (8.7)	.44	
	Outside Nordic country	11 (10.9)	11 (10.7)	.96	
**Mother’s occupation, n (%)**					
	Employed	85 (84.2)	81 (78.6)	.31	
	Student	1 (1)	2 (1.9)	.58	
	On leave or maternal leave	4 (4)	8 (7.8)	.25	
	Unemployed	9 (8.9)	10 (9.7)	.85	
	Do not know	6 (5.9)	7 (6.8)	.80	
**Father’s occupation, n (%)**					
	Employed	72 (71.3)	72 (69.9)	.83	
	Student	1 (1)	2 (1.9)	.57	
	On leave or paternal leave	4 (4)	7 (6.8)	.37	
	Unemployed	9 (8.9)	10 (9.7)	.85	
	Do not know	16 (15.8)	16 (15.5)	.95	
Never consumed alcohol, n (%)		14 (14)	11 (10.7)	.49	
Consumed alcohol to the level of intoxication, n (%)		65 (64.4)	79 (76.7)	.05	
Age of onset (years), mean (SD)		13.7 (2.01)	13.7 (2.14)	.98	
Age of first intoxication (years), mean (SD)		14.8 (1.36)	14.8 (1.71)	.82	
Center for Epidemiological Studies Depression Scale scores, mean (SD)		27.0 (12.82)	25.8 (12.61)	.51	
Coping With Parents Abuse Questionnaire scores, mean (SD)		48.57 (9.46)	48.70 (10.11)	.94	
Alcohol Use Disorders Identification Test scores, mean (SD)		2.49 (2.90)	2.69 (2.75)	.61	
**Ladder of Life scores, mean (SD)**					
	Past	4.51 (2.43)	4.40 (2.37)	.73	
	Present	5.36 (2.02)	5.20 (1.85)	.58	
	Future	6.80 (2.21)	6.85 (2.19)	.87	

^a^Significance levels calculated by Pearson chi-square statistics for categorical variables and 2-tailed *t* tests for continuous variables.

### Treatment Effects

For the primary outcome, coping behavior (CPAQ), we found a small but inconclusive treatment effect in favor of treatment at both 2 (t_1_) and 6 (t_2_) months (Cohen *d*=–0.05 at both t_1_ and t_2_). For the secondary outcome, alcohol use (AUDIT-C), we found a treatment effect in that the intervention group scored 38% less than the control group on the continuous part (ie, drinking when it occurred) at t_1_ and 46% less at t_2_. Regarding depression (CES-DC) and life status (LoL), all between-group comparisons of treatment effects were inconclusive at both follow-up time points (Table 2).

**Table 2:** Estimated treatment effects at 2-month (t_1_) and 6-month (t_2_) follow-ups.

Outcome		2-month follow-up (t_1_)				6-month follow-up (t_2_)		
		Treatment effect	Cohen *d* (95% CI)	*P* value	Treatment effect		Cohen *d* (95% CI)	*P* value
CPAQ^a^		–0.44	–0.05 (–0.32 to 0.23)	.74	–0.44		–0.05 (–0.32 to 0.23)	.75
CES-DC^b^		–0.54	–0.04 (–0.31 to 0.23)	.76	–2.61		–0.21 (–0.48 to 0.07)	.11
**LoL^c^**								
	Past	–0.37	–0.16 (–0.44 to 0.12)	.27	0.11		0.05 (–0.23 to 0.33)	.75
	Present	–0.24	–0.12 (–0.43 to 0.18)	.43	0.12		0.06 (–0.25 to 0.37)	.69
	Future	–0.49	–0.22 (–0.50 to 0.06)	.12	–0.18		–0.08 (–0.36 to 0.20)	.56
**AUDIT-C^d^**			Ratio	N/A^e^			Ratio	N/A
	Drinking (continuous)	–0.47	0.62 (0.44 to 0.88)		–0.62		0.54 (0.38 to 0.76)	
	No drinking (binary)	0.71	1.97 (0.25 to 19.52)		1.19		3.07 (0.40 to 40.79)	

^a^CPAQ: Coping With Parents Abuse Questionnaire.

^b^CES-DC: Center for Epidemiological Studies Depression Scale.

^c^LoL: Ladder of Life.

^d^AUDIT-C: Alcohol Use Disorders Identification Test.

^e^N/A: not applicable.

### Missing Data

In contrast to the ITT analyses, the sensitivity analyses showed that the treatment group, averaged over the levels of dropout, scored higher (ie, a negative effect) on the main outcome, coping behavior (CPAQ), at t_1_ (2.44; *P*=.20). However, the results remain inconclusive.

### Dose-Response Effects

We did not find any evidence for greater involvement in the program being linked to improved outcomes with regard to coping behavior.

## Discussion

### Overview

We did not find any support for the primary hypothesis: the intervention was not superior to the control condition with regard to coping behavior. Inconclusive results with small effect sizes were observed at both follow-up time points. However, for the secondary outcomes, we found that those in the intervention group who drank alcohol drank approximately 40%-50% less than those in the control group at both follow-ups. These results corroborate previous findings on the precursor face-to-face ICAIP intervention program, demonstrating that participants who received a combined alcohol and coping intervention reported superior outcomes with regard to alcohol-related outcomes compared to participants in the other 2 study arms, who received only a coping or alcohol intervention [41,42]. In contrast to this study, Hansson et al [42] found that all groups improved their coping skills, although the between-group comparisons were inconclusive and the improvements were maintained over time. These differences could be explained by the different settings in which the precursor program was provided (ie, face-to-face to young adults in a university setting), whereas this study targeted young people (15-19 years of age) through a web-based digital intervention. Additionally, the poor adherence in this study may explain the absence of primary results favoring the intervention group. In a recent study, parents without alcohol problems were recruited to participate in a randomized trial evaluating the web-based SPARE (Supportive Parenting and Reinforcement) program to improve children’s mental health and reduce coparents’ alcohol use. In line with our study, the authors did not find the primary outcome of the SPARE program to be superior to that of the active control group (which received written psychoeducation); however, both groups reported decreased coparental alcohol consumption [38].

Considering that approximately 3600 children in 2022 participated in various forms of support provided by Swedish municipalities [23], our recruitment activities reached a large number of eligible individuals, pointing to the potential of finding these children on these platforms. There were unexpectedly high levels of depression among the participants in this study. Although the intervention did not target depressive symptoms per se*,* there was a trend for the intervention group to have decreased depression levels compared to the control group. A large proportion of participants had symptoms of severe depression, which may have aggravated their capacity for improvement at follow-up [28,67]. Targeting dysfunctional coping patterns could affect an individual’s perceived mental health, and studies have shown that healthy coping strategies positively affect depression and anxiety in a positive way [68]. Using dysfunctional coping strategies, such as negative self-talk and alcohol consumption, can lead to depressive symptoms [69]. Targeting these symptoms in the context of healthy and unhealthy coping strategies may be a viable route to fostering appropriate coping strategies that work in the long run. Given that the young people who were reached by the intervention in this study displayed high levels of depression, future interventions for this group should include programs targeting depressive symptoms.

Almost 37% (37/101) of the intervention group did not log into the intervention at all, and only 24% (24/101) of the intervention group participants completed all parts of the program. The fact that a high proportion of the participants had symptoms of severe depression could explain the low adherence. Another reason could be that the initial film-based lectures were too long to maintain the participants’ attention, as the lectures ranged from 8-15 minutes. Yet a final reason could be that we had a 1- to 2-day break built into the intervention, and for unknown reasons, some participants did not log back into the intervention. However, we did not find a dose-response relationship indicating favorable outcomes for those who completed more of the program content. High levels of attrition are not uncommon in self-directed programs such as the one in this study; for example, in a study on a smoking cessation intervention, 37% of the participants never logged into the platform [70], and in a self-directed intervention for problem gamblers, a majority dropped out after 1 week and none completed the entire program [71]. Increased intervention adherence is a priority when developing new digital interventions, particularly for young people. One method is to use more persuasive technologies, such as primary tasks, dialogue, and social support [72]. Considering children whose parents have mental disorders, Grové and Reupert [73] suggested that digital interventions should include components such as providing information about parental mental illness, access to health care, genetic risk, and suggestions for how children might initiate conversations with parents who have the illness. These suggestions should be considered in future studies on interventions for youths whose parents have substance use problems. Representatives of the target group and other relevant stakeholders should also be involved in coproducing new interventions to increase the probability of developing more engaging programs [74]. Moreover, one cannot expect study participants to return to the program more than once, and for the sake of adherence, briefer interventions should not encourage participants to log-out for a break. To keep adherence at an acceptable level, similar future interventions for this target group should also consider having symptoms of severe depression as an exclusion criterion [28,67]. Further, to improve adherence, strategies of coproduction could be used where all stakeholders, including the target group, are involved in intervention development [75]. Other important factors identified to improve adherence to digital interventions are to make the content relatable, useful, and even more interactive [76]. Those participants who have symptoms of severe depression should be referred to other appropriate health care. Finally, it is probably beneficial to develop shorter psychoeducative film-based lectures than ours, lasting up to 15 minutes. Future self-directed digital interventions targeting this population should, therefore, focus on a very brief and focused intervention, which, based on theory, has the potential to foster healthy coping behaviors that can lead to an increased quality of life and improved mental health for this group of young people.

Another concern for future projects would be to use a data-driven approach during the program development phase, where A/B testing can be used to test different setups of the program to highlight which setup works best. Another aspect that must be considered is the fast-changing world of technology, where young people are exposed to an infinite number of different apps that grab their attention, which also calls for interventions to be short and to the point. Furthermore, if the program is to spread and become generally available, one must consider that keeping the program alive for a longer period will require funding and staffing for both product management and technical support.

### Strengths and Limitations

This study had several strengths. First, Alcohol and Coping is a web-based intervention program, and it appears as if the internet is a particularly promising way to provide support to adolescents growing up with parents with alcohol problems because it offers an anonymous means of communicating and makes intervention programs readily accessible [25]. Our recruitment strategies reached a considerable number of interested and eligible individuals, demonstrating the potential for recruiting through social media and other web platforms. Additionally, this program is one of the first brief web-based interventions aimed at adolescents with parents with alcohol-related problems. We used the CAST-6, which has been validated among Swedish adolescents [47], to screen eligible participants. Another strength is that the intervention program involved personalized, tailored feedback in the form of prewritten automatic messages and therapist-written personalized feedback, both of which have proven to be important components of web-based interventions aimed at adolescents [77,78]. Finally, this study evaluated the effectiveness of the Alcohol and Coping program using a randomized controlled trial design, which is considered the strongest experimental design with regard to allocation bias.

This study had some limitations. First, the design with a passive waitlist control group and an active intervention group, both unblinded to study allocation, may have resulted in biased estimates of treatment effects. Intervention adherence was low, and most of the study participants had symptoms of depression, where 55% (87/158) had symptoms of severe depression. This may have contributed to the small and overall inconclusive effects on the primary outcomes of this study. Many digital interventions have problems with low adherence, and in a review by Välimäki et al [79], some studies reported adherence rates as low as 10%. A vast proportion of the study participants were women, making the findings difficult to generalize to men. However, another limitation concerns selection bias and external validity. We recruited study participants through social media and other relevant websites containing health-related information, including information about parents with alcohol-related problems. It is, therefore, possible that the study population can be classified as “information-seeking” adolescents, who may have different personality traits relative to other adolescents in the same home situation. Additionally, as an inclusion criterion was having ready access to computers and the internet, it is possible that participants belonging to a lower socioeconomic class were underrepresented in the study. It should also be noted that the data presented here were collected approximately 10 years ago. However, we believe our findings make an important contribution to the field since, like our intervention, many recent web-based interventions use strategies of psychoeducation, films, exercises, questions, and feedback. Further, the number of web-based interventions for this target group remains scarce in the literature, which underscores the need for future research. Finally, the study was powered to detect a medium effect size. However, given the small effect sizes detected in this study, it is plausible that too few participants were recruited to detect differences between the groups.

### Implications for Practice

Although growing up with parents who have alcohol problems per se is not sufficient for developing psychosocial disorders, many children need support to manage their situation. Therefore, it is difficult to recruit children to support these groups. In Sweden, not even 2% of all children growing up with parental alcohol problems attend face-to-face support groups provided by municipalities.

Offering support through web-based intervention programs seems particularly attractive to adolescents whose parents have alcohol-related problems. To date, evidence for such programs is scarce, and there is an urgent need to develop and evaluate digital interventions targeting this group of adolescents. This study makes important contributions to this novel field of research. The results provide insight into effective strategies for delivering intervention programs to children of parents with substance abuse issues, highlighting the potential for digital interventions to reach a vulnerable, hard-to-reach group of adolescents. Our findings underscore the need to develop more engaging interventions in coproduction with the target group.

### Conclusions

We found that a digital therapist-assisted self-management intervention for adolescents whose parents have alcohol use problems contributed to a reduction in the adolescents’ own alcohol consumption. This result highlights the potential for digital interventions to reach a large, vulnerable, and hard-to-reach group of adolescents with support efforts. Findings were inconclusive for all other outcomes, which may be attributable to low adherence. This points to the need for future research on developing more engaging digital interventions to increase adherence among adolescents.

## Appendix

Multimedia Appendix 1 CONSORT-eHEALTH checklist (V 1.6.1).

## Abbreviations

AUDIT-C: Alcohol Use Disorders Identification Test
brms: R-package Bayesian regression models using Stan
CAST-6: Children of Alcoholics Screening Test
CES-DC: Center for Epidemiological Studies Depression Scale
CPAQ: Coping With Parents Abuse Questionnaire
GLMM: generalized linear mixed model
ICAIP: Individual Coping and Alcohol Intervention Program
ITT: intention-to-treat
LMM: linear mixed models
LoL: Ladder of Life
SPARE: Supportive Parenting and Reinforcement program
